# Supplementary material for: Chemical Composition, In Vitro Bioaccessibility and Antioxidant Activity of Polyphenolic Compounds from Nutraceutical Fennel Waste Extract
Source: Molecules. 2021 Mar 31;26(7):1968. doi: 10.3390/molecules26071968 (PMC8037122; doi:10.3390/molecules26071968)
Supplement: Supplementary file 1 [file molecules-26-01968-s001.pdf]

Supplementary materials:

Table S1. Correlation between TPC and data obtained by the DPPH, ABTS, and FRAP tests. The correlation coefficients were evaluated by using Pearson's method.

**Assay**	**Gastric Stage**	**Duodenal Stage**	**Pronase Stage**	**Viscozyme Stage**
	R^2	R^2	R^2	R^2
DPPH	0.99	0.99	0.98	0.99
ABTS	0.98	0.98	0.99	0.99
FRAP	0.98	0.98	0.98	0.98

Table S2. Stock solutions composition

		**SSF (pH 7)**		**SGF (pH 3)**		**SIF (pH 7)**	
Salt solution	Stock concentration (mol/L)	mL of Stock added to prepare 0.4 L (mL)	Final salt concentration in sample (mmol/L)	mL of Stock added to prepare 0.4 L (mL)	Final salt conc. in sample (mmol/L)	mL of Stock added to prepare 0.4 L (mL)	Final salt conc. in sample (mmol/L)
KCl	0.5	15.1	15.1	6.9	6.9	6.8	6.8
KH_2PO_4	0.5	3.7	1.35	0.9	0.9	0.8	0.8
$NaHCO_3$	1	6.8	13.68	12.5	25	42.5	85
NaCl	2	-	-	11.8	47.2	9.6	38.4
$MgCl_2(H_2O)_6$	0.15	0.5	0.15	0.4	0.12	1.1	0.33
$NH_4(CO_3)_2$	0.5	0.06	0.06	0.5	0.5	-	-

SSF: simulated salivary fluid; SGF: simulated gastric fluid gastric; SIF: simulated intestinal fluid.
